# Image quality evaluation of imaging skins, a novel stretchable X-ray detector for intraoperative tumour imaging

**DOI:** 10.1038/s41598-025-96768-z

**Published:** 2025-04-11

**Authors:** Solène Dietsch, Harry Allan, Lukas Lindenroth, Robert Moss, Agostino Stilli, Danail Stoyanov

**Affiliations:** 1 Wellcome/EPSRC Centre for Interventional and Surgical Sciences, University College London, London, W1W 7EJ UK; 2 Department of Medical Physics and Biomedical Engineering, University College London, London, WC1E 6BT UK; 3 X-ray Microscopy and Tomography Lab, The Francis Crick Institute, London, NW1 1AT UK; 4 School of Biomedical Engineering & Imaging Sciences, King’s College London, London, SE1 7EU UK

**Keywords:** X-ray detector, Soft materials, Stretchable sensor, Scintillator, Image quality, Biomedical engineering, Surgical oncology, Cancer imaging, X-rays

## Abstract

This paper presents the development of a novel X-ray detector composed of silicone elastomer and GOS:Tb, which we refer to as *imaging skins*. These detectors were integrated into a custom X-ray system to convert radiation into visible light. Our study focused on how fabrication parameters such as thickness and concentration impact sensor linearity, considering their potential application directly on the skin or organs to identify tumour margins during surgery. In addition, we examined how the stretching capabilities of these detectors influenced the image quality. Our imaging detection stack demonstrated consistent linearity across various fabrication parameters with the coefficient of determination ($R^2$) more than 0.99998, showing that the silicone elastomer does not affect the conversion of the X-ray into light. We achieved a spatial resolution of 1.16–1.42 lp/mm at 10% of the Spatial Frequency Response using a 0.5-mm thick sensor. This study represents a first step towards integrating stretchable X-ray detectors into clinical settings, particularly on curved surfaces, to unlock their full potential in complex surgical configurations. It also highlights the need for a deeper understanding of the interactions between X-rays and detector materials to fully interpret the observed effects.

## Introduction

X-ray imaging has been a crucial tool in medical imaging since its discovery a century ago by Wilhelm Röntgen. There are two main methods for detecting X-rays and generating digital images. One method converts X-rays directly into electric charges using photodetectors. In contrast, the other method first uses a scintillator to convert X-rays into visible light, which is then detected by photodetectors.

Regardless of the type of sensor, X-ray detectors have traditionally been designed to be rigid and flat^1,2^. However, these detectors are restricted in their ability to image 3D objects^3^, lack the flexibility required to conform to complex structures, and often require complex and costly fabrication processes. In addition, they typically employ brittle materials and scintillators made of heavy substances^4^, making X-ray systems large and expensive^1^.

Nevertheless, the continuous discovery of new scintillators, particularly those made from organic materials with a high light yield, presents an opportunity to develop flexible and stretchable X-ray detectors^1,5–7^. Recent advances in scintillator materials have led to the development of flexible X-ray detectors. Although studies are mainly directed towards the discovery of new scintillators^8–10^, some research teams have started to develop the concept of flexible or stretchable X-ray detectors^3,11,12^.

In this article, we call these stretchable X-ray detectors *Imaging skins* due to their conceptual similarity to electronic skins (E-skins)^13–16^. Unlike E-skins, the imaging skins contain no embedded electronics but share the objective of being wearable and directly applicable to the skin or organs of patients, while providing images of the underlying tissues. Compared to flat X-ray detectors, imaging skins can be stretched and adapted to complex anatomical structures without requiring compression. Their close proximity to the imaged tissue may enhance resolution, thanks to both the reduced distance and their ability to conform to intricate organ shapes. This proximity could also allow for a lower dose to achieve high-quality images, as attenuation and noise are minimised with reduced absorption and scattering from distant detectors. Furthermore, these innovative sensors can be handled at room temperature^6,9^, potentially transforming the large-scale fabrication process^17^ and enabling applications in various fields, such as intraoperative medical imaging.

**Fig. 1:**
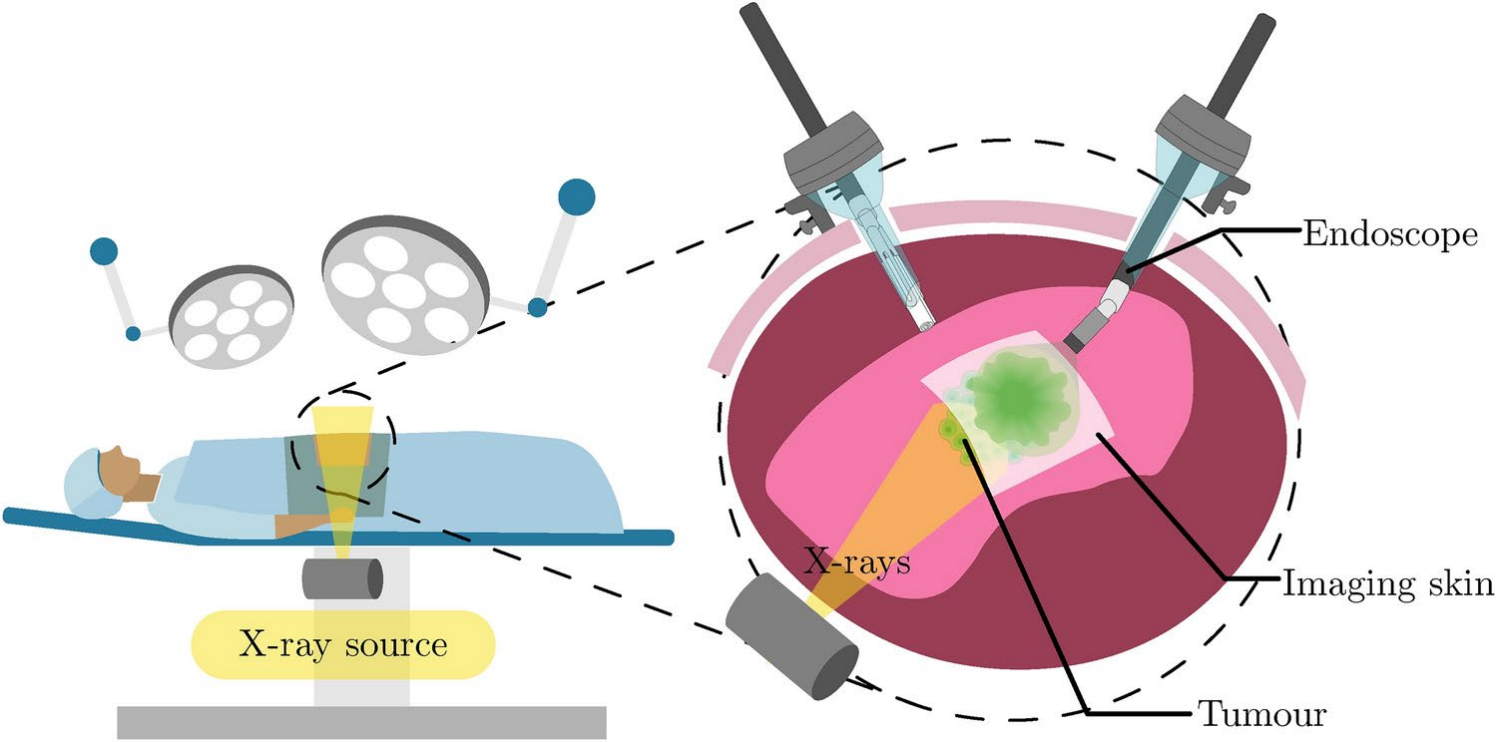
Imaging concept. An imaging skin is inserted through a trocar port and placed on the surface of the organ. The X-ray source is activated, and the scintillation is captured by an endoscope. Illustration created using Adobe Illustrator.

Minimally invasive surgeries (MIS) face additional challenges due to the complexity of accessing the surgical site; the incision port restricts both visual inspection and palpation, whether direct by hand or indirect through instruments. As a result, surgeons rely largely on pre-operative imaging or post-operative assessments to select and remove targeted tissue. Commonly used planning and monitoring techniques include Computed Tomography (CT), Magnetic Resonance Imaging (MRI), and Ultrasound^18,19^. However, these techniques have limitations. CT and MRI are bulky and impractical for intraoperative use; CT exposes patients to high radiation doses and lacks real-time feedback, while MRI provides excellent soft-tissue contrast but is costly and slow^18,19^. Ultrasound is real-time and portable but is highly operator-dependent and has limited resolution^18,19^. Emerging imaging techniques show great promise but have yet to fully mature as a standard of care^19,20^. For instance, surgical spectral imaging and optoacoustic imaging lack continuous feedback^21^, while fluorescence-guided surgery does not provide subsurface information^20^.

Fluoroscopy, an X-ray-based imaging method, offers an intraoperative option for tumour detection, providing real-time video imaging. Advances in contrast agents that accumulate in tumour tissues have further enhanced its utility^22^. However, its resolution remains in the cubic-millimetre range, which falls short of the 1–100 μm resolution achieved by other imaging solutions. Additionally, fluoroscopic systems are large and occupy substantial space in the surgical theatre, presenting logistical challenges^19^. The equipment size, high cost, and risk of clinician radiation exposure also limit its adoption compared to other standard imaging techniques^19^.

Imaging skins could transform intraoperative X-ray imaging by converting X-rays into visible light in real time and enabling imaging over large surface of tissues. Their stretchability allows easy insertion through a trocar port during MIS, making it possible to image areas that are otherwise difficult to access and providing deeper in-body imaging. We envision deploying imaging skins directly onto targeted organs, as illustrated in Fig. 1. This approach could reduce the required exposure dose and improve resolution, as X-ray photons would encounter less attenuation from surrounding tissues and reduced dispersion compared to distant detectors.

In contrast to studies presenting a similar sensor concept, but primarily focused on evaluating scintillator performance, this study aims to assess the medical imaging potential of stretchable X-ray detectors^3,8–11^. We introduced an imaging stack for producing digital images and analysed how specific fabrication characteristics of the imaging skin impact the sensor performance. Our primary focus was to ensure that our optical imaging stack could obtain X-ray images and to evaluate the impact on imaging quality in this context, with particular emphasis on the stretchability of the detectors. Subsequently, we imaged various objects to confirm that we could produce high-quality X-ray images with the imaging skins.

## Materials and methods

### X-ray imaging setup

**Fig. 2:**
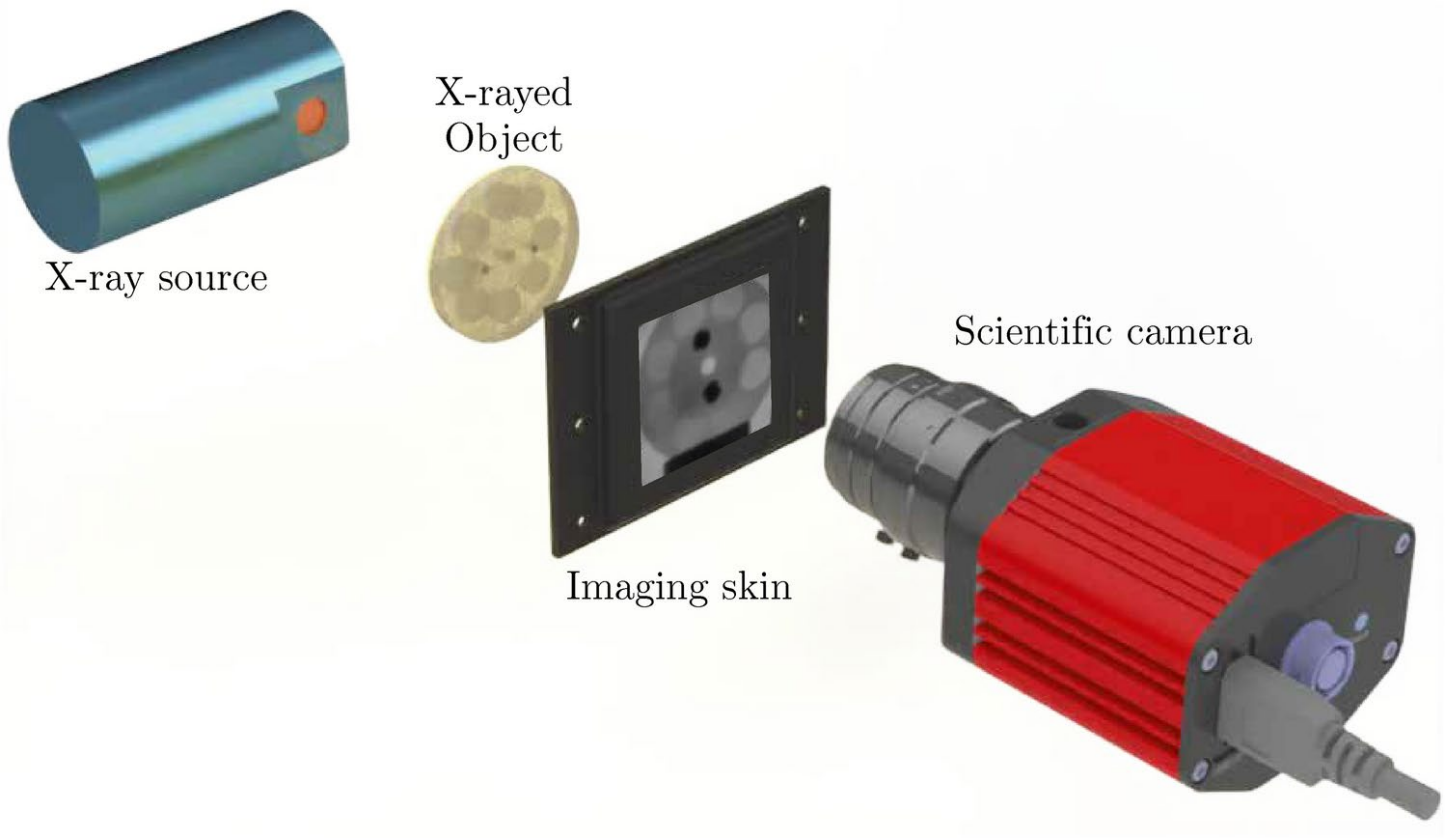
Schematic of the experimental setup. From left to right, the X-ray source, the X-rayed object, the imaging skin, and the scientific camera. X-rayed objects are intercalated between the X-ray source and the imaging skin, as close to the imaging skin as possible.

To assess the performance of the imaging skins, we constructed a custom X-ray detection system, as illustrated in Fig. 2. The system comprises an X-ray source, the imaging skins serving as the radiation converter, and a scientific camera to record the light emission from the imaging skin. Any scanned objects in this study were intercalated between the X-ray source and the imaging skin, as illustrated in Fig. 2. The objects were positioned as close to the imaging skin as technically possible.

#### X-ray source

In the experiments presented, X-rays were generated using a fixed THX160 1055 (THALES, Vélisy, France) monopolar X-ray tube with a 5.5 mm focal spot in fluoroscopy mode, controlled by a Gulmay high-voltage generator and MP1 controller. The tube current and tube voltage were automatically set through Matlab R2023b (THE MATHWORKS LIMITED, Cambridge, United Kingdom) by sending commands through the connected COM port. The inherent filtration of the source is 1 mm Be with an anode angle of 21°, as summarised in Table 1.

In our study, we sought to confirm the detectability of light emitted by our X-ray converter within X-ray energy ranges typical of medical imaging and its potential for soft tissue imaging. Therefore, we selected three tube settings derived from quality standards^23^, covering a range of X-ray applications such as neonatal, pediatric, extremity, and general radiography^24^. However, experiments were limited to tube voltages above 40 kVp, as the X-ray source became unstable below this threshold and could not provide a consistent spectral beam, thus preventing tests at voltages more suitable for soft tissue imaging.

We adjusted the tube current to match those used in the O-Arm (Medtronic, MN, US), a commonly used fluoroscopy system (Table 1). All experiments were conducted without additional filtration. Consequently, while the data provide valuable insight into the behaviour of our detector, caution is advised when interpreting it, as it may not be directly comparable to other digital sensors that comply fully with Radiation Quality standards used in medical radiology.

**Table 1:** X-ray tube specifications.

X-ray tube settings			
Tube voltage (kVp)	40	50	70
Tube current (mAs)	7.2	7.8	9.2

Additional parameters			
Focal spot size (mm)	5.5		
Target material	Tungsten		
Target angle (°)	31		
Inherent filtration (mm Be)	1		
Additional filtration	None		

#### Dosimeter

We measured the effective dose reaching the imaging skin with the RaySafe Solo R/F dosimeter (Unfors RaySafe AB, Sweden), specifically designed to measure high dose rates in medical X-ray imaging. To calculate the air kerma ($A_k$), we averaged the dose rate (*d*) over five measurements for each setting of the X-ray tube, recorded in mGy/s, and multiplied it by the exposure time *t* (s): $A_k = d \times t$.

#### Imaging skins

The imaging skins were fabricated by mixing Sylgard 186 elastomer (Dow, MI, U.S.) and gadolinium oxysulfide:terbium (GOS:Tb) powder (Phosphor Technology, England), a commercially available phosphor (see Supplementary Fig. S1)^25^. The mixture was then moulded into 50 × 50 mm square skins using 3D printed moulds made of clear resin (Formlabs, MA, U.S.). A wide range of fabrication parameters was tested to evaluate the impact of imaging skin design, varying the substrate-to-phosphor ratio and thickness. The resulting specimens are detailed in Table 2.

**Table 2:** Imaging skins fabrication parameters.

Parameter	Value 1	Value 2	Value 3
Thickness (mm)	0.5	2	5
Ratio (substrate-to-phosphor)	4:1	2:1	1:1

#### Camera

To capture the scintillation, we employed a 5.0 MP monochrome CMOS camera, specifically the CS505MU Kiralux Compact Scientific Digital Camera (Thorlabs, Inc., NJ, USA). The sensor consists of 1624 × 1224 elements with 3.45 μm pixel pitch. Its quantum efficiency is optimal around 480-580 nm, corresponding to the peak emission spectrum of the GOS:Tb (see Supplementary Fig. S2). It is complemented by a lens with a fixed focal length of 12 mm, resulting in an effective image pixel size of 51 μm (see Supplementary Fig. S3). To ensure a precise focus of the camera onto the imaging skin, we replaced it with a chequerboard calibration target. This target consists of 12 × 9 × 5 mm squares. Subsequently, we manually adjusted the lens settings to achieve the sharpest image quality. We controlled the camera settings using the ThorCam SDK through our custom Python software. The exposure time was set to 100 ms, with a gain of 5 dB. This configuration was determined through manual experimentation to ensure scintillation could be visually detected at low X-ray doses (40 kVp, 7.2 mAs tube voltage) without saturating the image sensor at higher settings (120 kVp, 11.7 mAs). In addition, we set a frame buffer size of 10 images. Table 3 summarises the parameters used in this setup. Each frame used here is an average of the buffered frames, reducing noise from X-ray photons detected by the camera sensor and other inherent sources^26^.

**Table 3:** Camera and experiment settings.

Settings	Value
Nb averaged frames per data	10
Exposure (ms)	100
Gain (dB)	5
Lens focal length (mm)	12
Camera sensor pixel pitch (μm)	3.45
Effective image pixel pitch (μm)	51
Detector-to-source distance (m)	1.01 (flat variation)
	0.56 (stretching)

### Image quality

IEC standards facilitate the comparison of digital X-ray detectors with different materials and geometries, but they are typically designed for flat detectors with a fixed shape. As our goal was to assess how deformations affect detector performance, we selected and adapted the relevant image quality metrics from IEC 62220^27^ to suit our study.

#### Image pre-processing

**Fig. 3:**
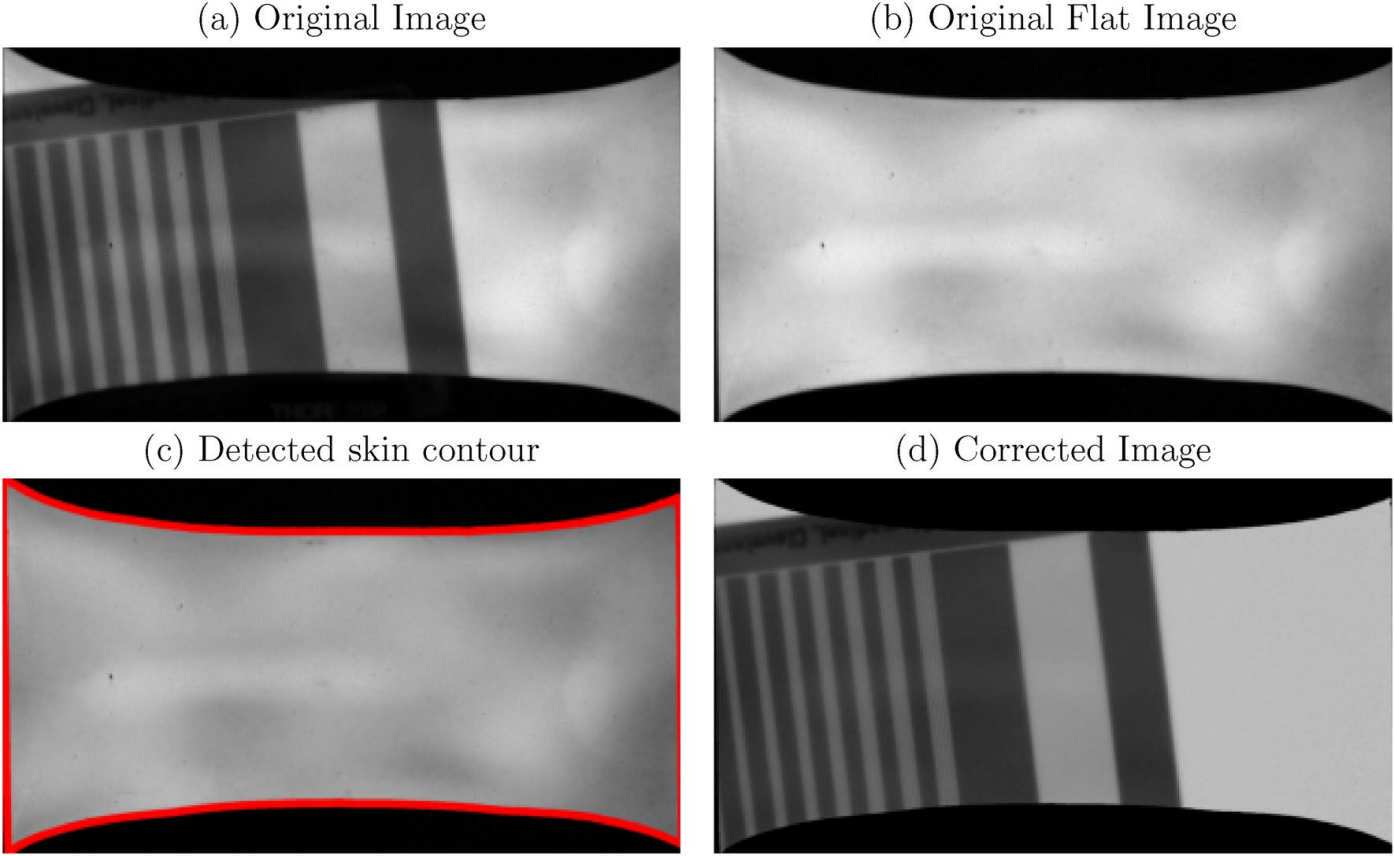
Pre-processing correction. (**a**) Raw image. (**b**) Original flat image. (**c**) Detected imaging skin contour (in red) on the dark-field-corrected flat image. (**d**) Final corrected image.

We implemented dark-field subtraction and flat-field correction (Fig. 3a–d) for gain and offset correction using the standard method described in^28^ (Supplementary Algorithm 1):

1 $$\begin{aligned} I_{corrected} = \frac{\overline{I_{raw}}-\overline{I_{dark}}}{\overline{I_{flat}}-\overline{I_{dark}}}*m, m = \overline{\overline{I_{flat}}-\overline{I_{dark}}} \end{aligned}$$

With:

- $\overline{I_{raw}}$: Average of 10 raw frames. (Fig. 3a)
- $\overline{I_{flat}}$: Average of 10 flat field frames, acquired under the same conditions (same tube voltage/same integration time) but without the test object. (Fig. 3b)
- $\overline{I_{dark}}$: Average of 10 dark frames (no X-ray exposure).

Compared to regular, flat X-ray detectors, our detector can change position, shape, and size within the frame of the camera as even subtle movements could alter its placement. Therefore, we implemented automatic edge detection to find the detector contours in each image. Only pixels within these limits were used to perform flat-field correction, as depicted in Fig. 3c. The resulting correction was then applied to generate the final corrected image in Fig. 3d.

For each new imaging skin or repositioning, we performed skin contour detection at the highest experimental dose used: the maximum tube setting (70 kVp) and/or the longest exposure time (500 ms). The resulting ROI was then used to ensure data reliability, eliminating the need to re-detect the imaging skin border and associated issues during minimal scintillation.

#### Signal transfer property (STP)

The signal transfer property helps understand the relationship between the radiation dose reaching the sensor and its output^29^. This is evaluated by measuring the Mean Pixel Value (MPV) at varying radiation doses, or air kerma ($A_k$). The radiation dose can be adjusted by altering either the exposure time (ms) or the tube current (mA). In this study, we adjusted the exposure time due to its simplicity and greater reliability compared to tube current control. The MPV was calculated by averaging the pixel values across the entire detected Region-Of-Interest (ROI) of the imaging skins:

2 $$\begin{aligned} MPV(A_k) = \frac{1}{N} \sum _{(i,j) \in \text {ROI}} p_{ij}(A_k), \end{aligned}$$

where *N* is the total number of pixels within the masked ROI, $p_{ij}$ is the pixel value at a specific Air Kerma $(A_k)$ at the *i*, *j* pixel coordinates.

#### Edge spatial frequency response (e-SFR)

In this research, our objective was to understand the impact of embedding a scintillator into a stretchable substrate on imaging in both flat and stretched configurations on resolution. Therefore, we calculate the Edge Spatial Frequency Response (e-SFR), which assesses the resolution and contrast of the imaging system by quantifying its ability to reproduce fine details. Following IEC 62220^27^, we intercalated a 50 μm thick lead bar phantom between the X-ray source and the scintillator, carefully adjusting it with a small angle ($\alpha$; 2–7°) relative to the pixel matrix and placing it as close as possible to the imaging skins to limit blurring from magnification effects^28^. We manually selected the ROI, ensuring it included both dark and white signals in equal proportion. To calculate the e-SFR following IEC 62220^27^, we adapted the Matlab code and implemented the SFRMAT5 algorithm (Steps are detailed in Supplementary Fig. S4), as described in^30^. The reader should note that the ISO 12233^31^ standard employs the term e-SFR to account for the nonlinearity of the system, but is analogue to the presampled Modulation Transfer Function in IEC 62220^27^.

We report the complete e-SFR, SFR50, and SFR10, which correspond to the e-SFR at 50% and 10%. SFR10 is generally accepted as the threshold below which human vision cannot perceive finer details in an X-ray imaging system^32^.

### Experimental validation

Different experimental setups were created to understand the impact of various fabrication parameters of the sensor on imaging quality. The light yield of GOS:Tb is relatively high, measuring at 50,000 photons/MeV^7^. However, uncertainty persisted regarding its detectability using our custom X-ray detection system.

Therefore, the first experiment consisted of selecting the right camera parameters to ensure the emitted light was sufficient to obtain valuable X-ray images. Once we ensured that the signal was strong enough, we checked the linearity of the light signal by generating the STP curves at different tube settings reported in Table 1.

We then explored how the thickness of the imaging skin affected the imaging performance. The ability to conform to objects depends on the material properties and sensor shape. If the substrate is not flexible, like glass, the sensor will not conform regardless of its design. Similarly, a thick imaging skin may not conform even if the material is flexible. To recall, Sylgard 186 has a tensile strength of 305 psi (2.1 MPa or 21 kg/cm^2^) and a shore hardness of 24. It can sustain a 255% elongation before breaking^33^. Increasing the thickness of the scintillator leads to greater depth-of-field blur due to light scattering^26,34^. For this reason, as well as mechanical considerations, it is recommended that the scintillator be kept as thin as possible.

However, a thin scintillator may lack stopping power to capture all incoming energy, particularly at higher photon energies^35^ and can also be more difficult to fabricate and manipulate. Thus, there is a trade-off between selecting a thickness that maximises signal strength, and keeping it thin to enhance resolution and conformability.

We also investigated how the substrate-to-phosphor ratios affect the light output of the imaging skins because minimising the amount of phosphor is desirable due to its cost. To that end, we fabricated imaging skins with different thicknesses and substrate-to-phosphor mass ratios and compared their light output with different tube settings (see Table 1). We expected that increasing the skin thickness and the concentration of phosphor would result in greater light output. This occurs because increasing either the thickness or the phosphor concentration enhances the X-ray absorption efficiency of the detector. Which results in higher production of visible photons, as dictated by the light yield of the phosphor^36^.

As the name suggests, the advantage of using stretchable material is the ability to stretch it. While the application may not require stretching the silicone elastomer to its breaking point, understanding how changing its shape impacts imaging is important. For example, we envision the sensor will stretch to maintain contact with organs or skin surfaces, or adapt to internal movements caused by breathing or other natural organ motion.

We built a custom platform to analyse how stretching affects the sensor, stretching the imaging skin in 20% elongation increments (equivalent to 1 cm steps). The elongation is calculated as:

$$\begin{aligned} \text {Elongation} (\%) = \frac{L - L_0}{L_0} \times 100 \end{aligned}$$

where $L_0$ is the initial length of the imaging skin, and *L* is the stretched length. We measured the STP at multiple tube settings while the e-SFR was measured only at 70 kVp to evaluate the effect of stretching on the sensor. The reader should note that the results are specific to this experiment and not directly comparable to previous findings since the stretching platform was placed closer to the X-ray source, as reported in Table 3.

### Imaging

Again, the objective was to explore the capacity to obtain high-quality images with stretchable detectors. Additionally, we aimed to identify suitable materials for fabricating a medical phantom. To achieve this, we selected various objects made from materials commonly used in phantom fabrication and rapid prototyping, as mentioned in^37^.

## Results and discussion

### Signal transfer property (STP)

The optimal output for a digital X-ray detector is to achieve a linear or exponential relationship between the Detector Air Kerma ($A_k$) and Mean Pixel Value (MPV)^29^. This allows the pixel value to be subsequently correlated with specific absorption levels. For a model to be considered linear, it should be fitted to the experimental data and no experimental data point should deviate more than $2\%$ from the model fit^31^.

**Fig. 4:**
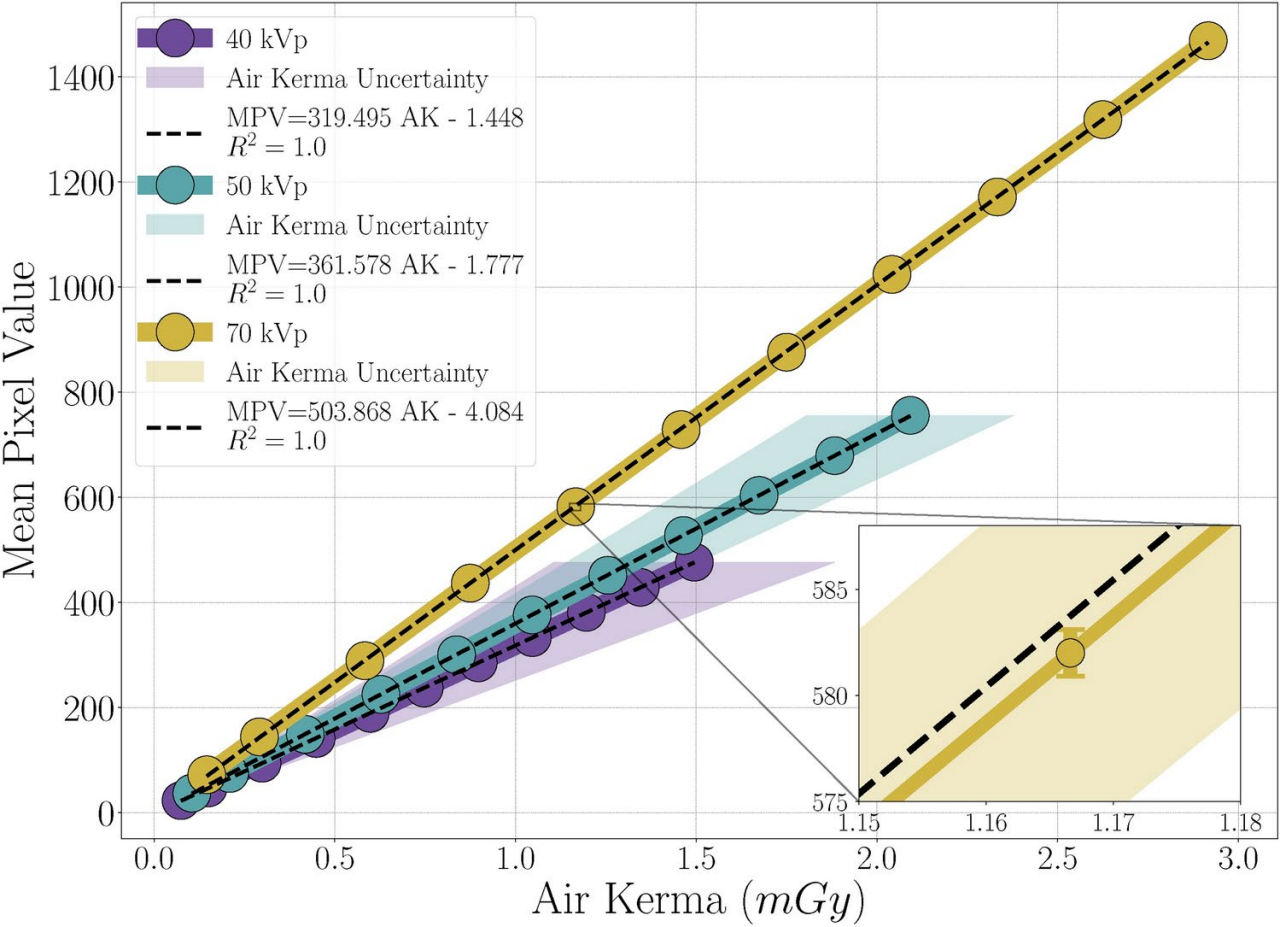
Signal transfer property. Relationship between the mean pixel value (MPV) and the Air Kerma ($A_k$) at different tube settings for the imaging skins with a 1:1 ratio and 0.5 mm thickness. In black, the corresponding linear regression.

As shown in Fig. 4, the STP curves exhibit near-perfect linearity with a coefficient of determination ($R^2$) greater than 0.99998 with all tube settings. This high degree of linearity can be attributed to the use of a scientific camera, which itself has a linear STP, and to the GOS:Tb phosphor, recognised for its consistent conversion of X-ray energy into visible photons. Furthermore, the variation in exposure time allowed for precise linear control of the dose reaching the detector. The air kerma uncertainty decreases with increasing tube voltage, possibly indicating reduced dosimeter effectiveness at lower energies. It could also come from source variations, but the strong linearity of the STP curves makes this unlikely. Additionally, the mean pixel value uncertainty remains minimal across all iterations, staying below 1.72. This is likely due to averaging over all detected pixels within the imaging skin, combined with each image being an average of 10 frames, further reducing variation in the final value for each iteration. Finally, we observe that the collected light signal increases as the dose increases. This can be explained by the linear nature of the GOS:Tb, which emits light as a function of the X-ray photon energy deposited in it^35^.

These results are very promising, demonstrating that the use of silicone elastomer as the substrate for the scintillator does not impact the linearity of X-ray to visible photon conversion. This finding has significant implications for the development of the next generation of X-ray sensors, confirming our substrate choice and potentially revolutionising the costly fabrication process of X-ray detectors^38–40^.

### Imaging skin thickness and substrate-to-phosphor ratio

**Fig. 5:**
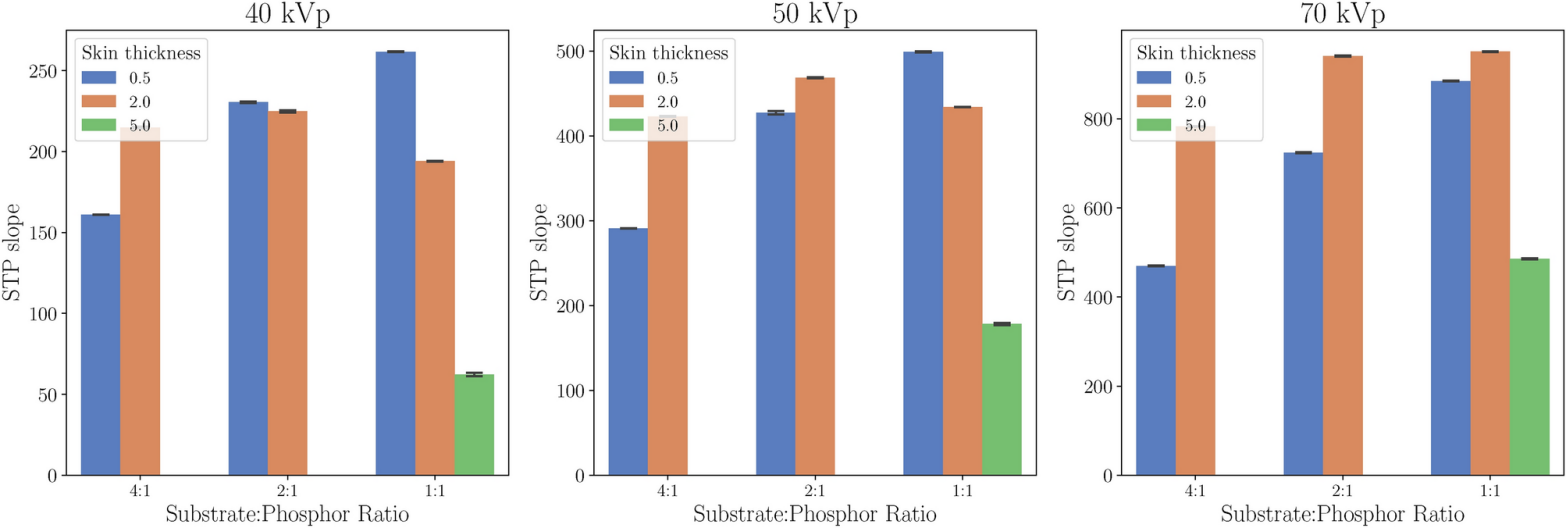
Bar plot of STP slopes at different phosphor concentration. Each subplot corresponds to a different tube voltage (40, 50, and 70 kVp). Blue bars represent 0.5 mm, orange bars 2 mm, and green bars 5 mm thickness. In black, the error bars represent the variation across iterations.

The STP curves exhibited linearity with respect to air kerma, as shown in Fig. 4, corresponding to the Imaging Skin with a 1:1 ratio and 0.5 mm thickness. Therefore, for clarity, each bar in Fig. 5 represents the calculated STP slopes for various skin thicknesses and phosphor ratios at specific tube voltages. The error bar, representing the variation across iterations, is plotted. As shown in Fig. 5, it remains below 1.93 for all tested prototypes.

We initially expected that increasing the thickness of the imaging skins and the phosphor concentration would result in a higher STP slope. While the concentration had a noticeable impact on the 0.5 mm imaging skins across all tube settings, this trend was not observed for the 2 mm-thick imaging skin at 40 kVp and 50 kVp, as shown in Fig. 5. Similarly, increasing the thickness of the scintillator did not consistently result in a stronger signal; in fact, the opposite occurred, particularly with 1:1 imaging skins across all tube settings.

Surprisingly, further verification revealed that a GOS:Tb scintillator with a thickness of less than 0.6 mm absorbs 90% of X-ray photons in the 20–40 keV range^35^. This suggests that X-ray photons cannot penetrate beyond a certain material thickness, meaning that any phosphor beyond this limit is not excited and, therefore, does not produce visible photons. A 1 mm thick scintillator absorbs 90% of the X-ray energy in the 20–70 keV range^35^, indicating that further increasing the thickness offers no significant advantage for signal acquisition with GOS:Tb. Consequently, a higher concentration of GOS:Tb would also increase the stopping power, further reducing the thickness beyond which the scintillator ceases to emit light.

Nonetheless, if this were the only phenomenon at play, the light output would reach a limit at a certain tube voltage and would not be affected by the thickness of the sensor. However, we observed that the 5 mm-thick imaging skin produces significantly less light output at 70 kVp. This may be explained by the fact that, beyond the thickness limit where X-rays can no longer penetrate, some of the light produced by the materials dissipates, potentially due to reabsorption by the imaging skin itself.

This suggests that further analysis is needed to fully explain these discrepancies. However, further optimisation would be necessary to determine the ideal thickness for a specific phosphor concentration, X-ray source, energy, and source distance. Given the number of variables involved, simulations could be beneficial for identifying the optimal parameters tailored to a specific application.

Therefore, contrary to our initial hypothesis, a thin scintillator film does not hinder X-ray detection at the tested energy levels. In fact, our experiment indicated that a thicker scintillator can result in lower light output for low-energy X-ray applications. Consequently, at this stage of imaging skin development, the trade-off in selecting the appropriate thickness will primarily depend on factors such as ease of fabrication and sensor handling.

**Fig. 6:**
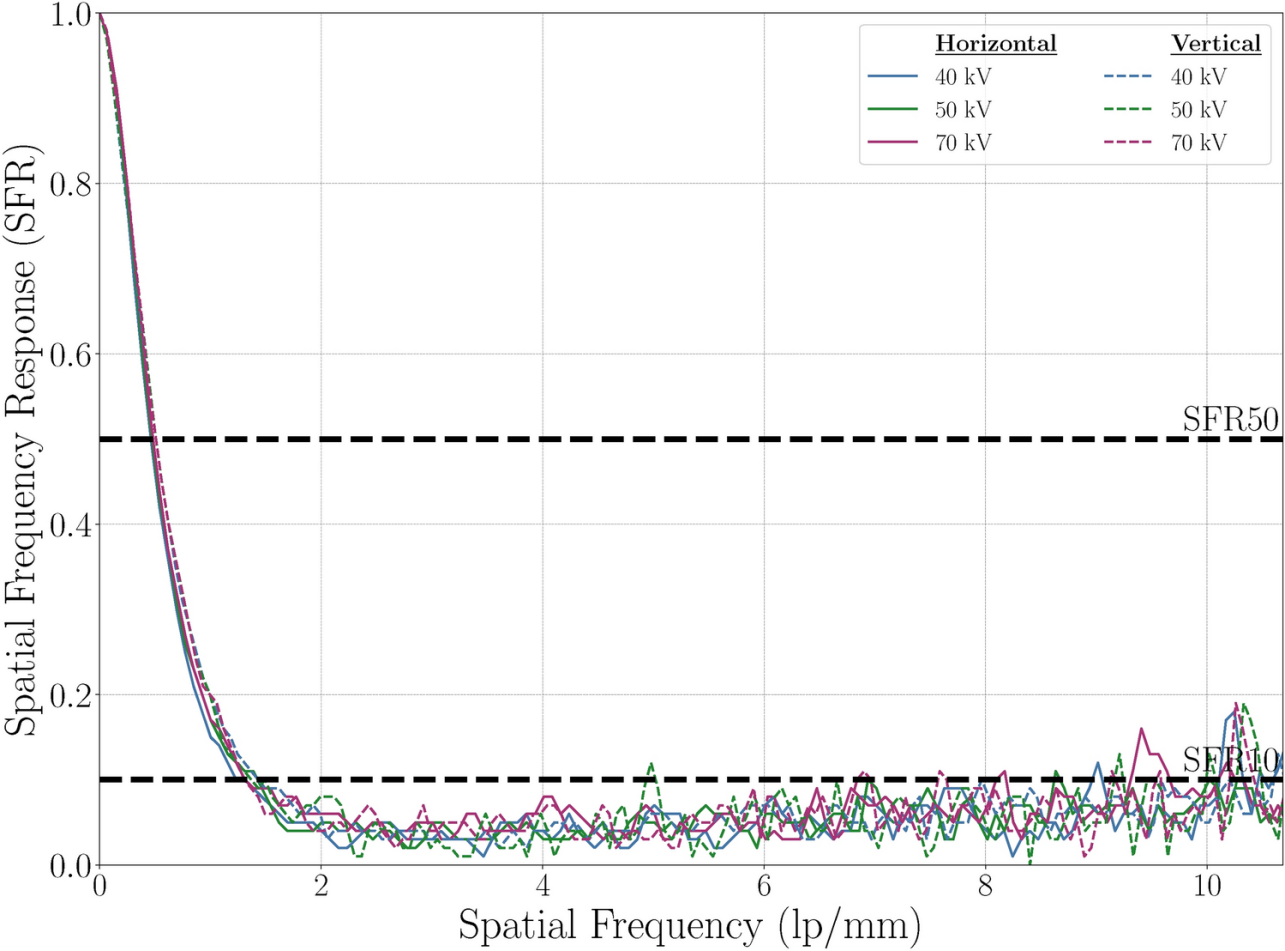
e-SFR analysis. This plot shows e-SFR results for various tube settings, with solid lines for horizontal e-SFR and dashed lines for vertical e-SFR.

### Edge spatial frequency response (e-SFR)

The e-SFR analysis yielded similar results for both horizontal and vertical orientations in all tube settings, as shown in Fig. 6. Horizontal SFR50 values ranged from 0.46 to 0.48 lp/mm, with SFR10 values between 1.16 and 1.20 lp/mm. Vertical SFR50 values ranged from 0.50 to 0.51 lp/mm, and SFR10 values were between 1.35 and 1.42 lp/mm. At this stage, the slight differences in these results remain unexplained but could be attributed to the anisotropy of the X-ray source or the imperfect alignment of elements within the imaging stack.

In general radiography, the limiting resolution (SFR10) for a detector is typically required to be above 3.0 lp/mm^34,41^, meaning that our detector resolution needs to be improved by approximately 150% for horizontal values and 110% for vertical values to meet this standard. However, we acknowledge that spatial resolution is also influenced by the X-ray source, such as the focal spot size or magnification. In our setup, the focal spot size (5.5 mm) is much larger than that of clinical fluoroscopy and radiography tubes, which may contribute to increased blur and limit direct comparison with clinical values.

Nevertheless, there is room for improvement. A finer X-ray source should be used to determine whether the source is the primary limiting factor or if the sensor itself requires further enhancement. The e-SFR could potentially be enhanced by using a thinner imaging skin, which would reduce light scattering and improve spatial resolution^26,34^. Additionally, optimising the scintillator thickness and phosphor concentration may further improve the e-SFR.

### Stretching experiment

**Fig. 7:**
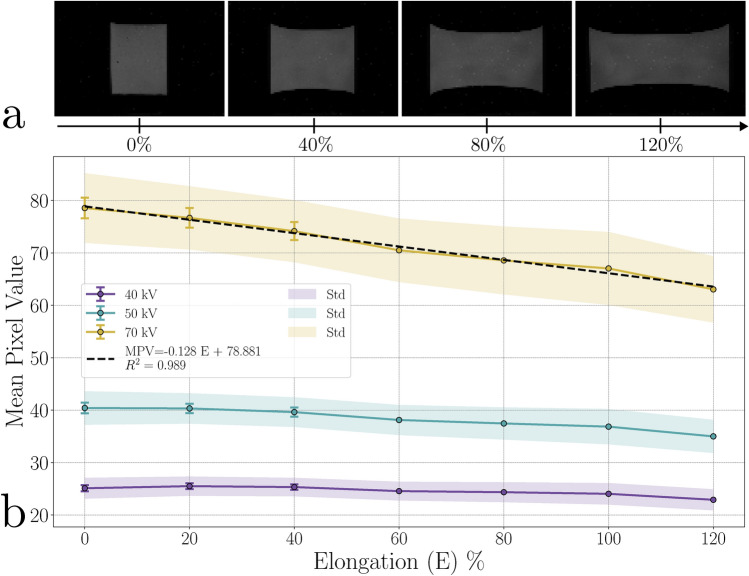
Stretching results. (**a**) Resultant images after stretching from 0 to 120% following dark field correction Tube voltage: 70 kVp). (**b**) Corresponding mean pixel values (MPV) at various elongations and tube voltages, with standard deviations calculated from multiple experiments (available for 0–40% elongation). The shaded area represents the standard deviation of pixel values within the imaging skin.

During stretching, we used a 0.5 mm thick imaging skin, which ruptured at 120% elongation–well below the 255% specified for PDMS. This discrepancy may arise from differences in the imaging skin’s geometry compared to the test specimen used to measure elongation at break, as well as variations in test conditions, such as elongation speed and room temperature, as standardised in ISO 37:2024^42^.

From Fig. 7, we observe that the MPV remains relatively stable at 40 and 50 kVp, showing a slight increase between 0% and 40% elongation, followed by a slight decrease beyond 20% and 30% elongation respectively. However, at 70 kVp, the MPV decreases linearly with increasing elongation, with an $R^2=0.989$.

Stretching affects the volume of the imaging skin in two opposing ways: it increases the surface area while simultaneously reducing its thickness. The evolution of thickness was estimated using image analysis, assuming volume conservation. Under this assumption, thickness decreased from 0.5 to 0.26 mm as elongation increased from 0 to 120%, as shown in Supplementary Fig. S5.

Based on this hypothesis, the scintillating particle distribution and stopping power remain unchanged as the volume remains constant. Therefore, the overall decrease can be attributed to the thinning effect of the imaging skin. While the stopping power remains unchanged, the reduced thickness significantly impacts the imaging skin’s efficiency in capturing radiation, particularly at 70 kVp, where this effect is linearly related to stretching.

At lower tube energies and small elongation, other phenomena may influence the response, as seen in the slight increase at 40 kVp and the stability between 0% and 20% elongation at 50 kVp, which appear less intuitive. A slight stretch can enhance transmittance by reducing self-absorption of scintillation light, an effect linked to decreased effective filler concentration, as reported by Oliveira et al.^12^, leading to higher optimal transmittance and an improved mean pixel value (MPV) until reaching an optimum.

Another possible explanation for the slight increase could be the surface warping of the imaging skin at lower elongations. This warping may cause certain areas to appear brighter due to their closer proximity to the camera detector, introducing shadowing effects from imperfect parallelism between the imaging skin and the camera.

To better understand the impact of stretching on pixel values, we analysed the evolution of pixel distribution during stretching, as shown in Supplementary Fig. S3. To quantify these changes, we examined variations in pixel intensity distributions using the Bhattacharyya distance after first removing the mean (Supplementary Fig. S6). This metric objectively measures how stretching modifies the emitted pixel distribution. The values remain below 0.35 across all tube energies, indicating that the overall distribution remains close to the initial state. However, results show a progressive deviation as elongation increases, as reported in Supplementary Fig. S7, with the distribution reaching a critical point near 120% elongation - just before the breaking zone - a trend also visually apparent in the images.

Additionally, the impact of stretching is examined in Supplementary Fig. S8. The zones were divided as a percentage of the detected imaging border to track their evolution. Except for areas near the border, where optical effects may play a role, all zones follow a similar trend, indicating that the redistribution of particles remains locally consistent across the entire imaging skin.

**Fig. 8:**
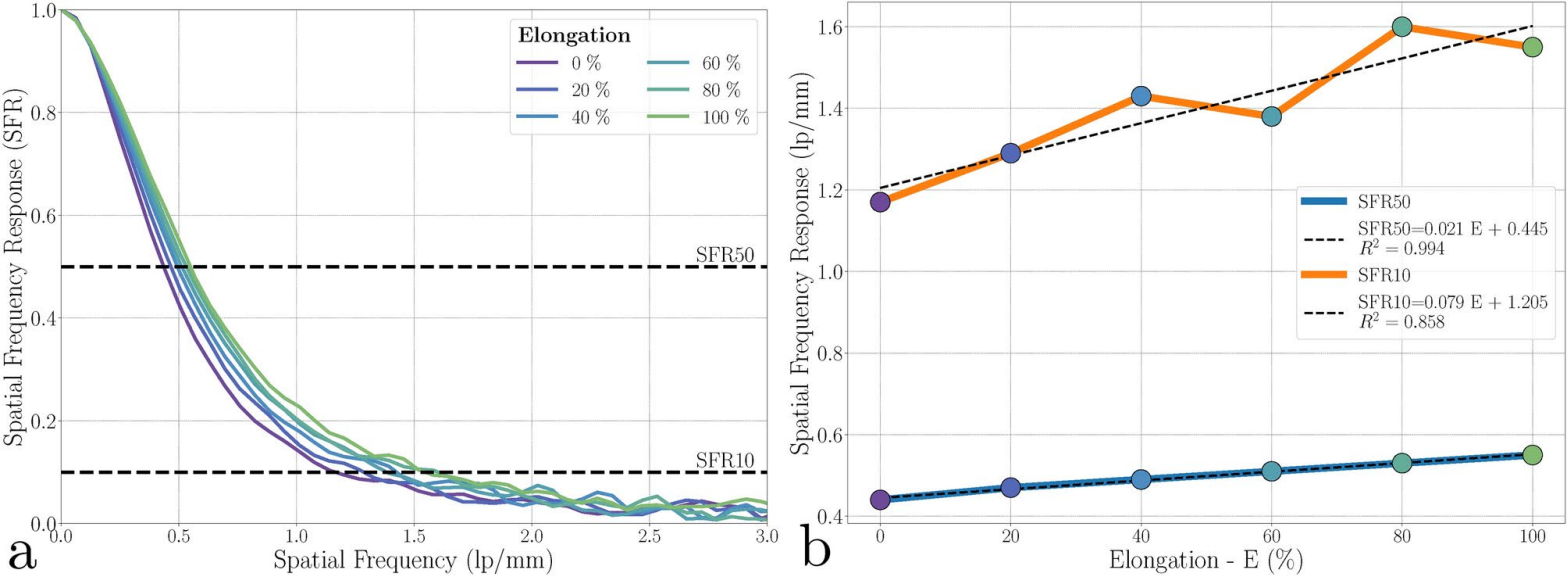
Spatial frequency response (e-SFR) evolution during stretching. (**a**) Horizontal e-SFR at each level of elongation. (**b**) Evolution of SFR10 and SFR50 as a function of elongation.

In Fig. 8a, we observe that the horizontal e-SFR follows a similar trend across all elongation levels. However, there appears to be an improvement in spatial frequency response with increasing elongation. To better understand this trend, we plotted the evolution of SFR50 and SFR10 at the different elongation steps in Fig. 8b. The SFR50 ranges between 0.44 to 0.55 lp/mm, showing an overall increase. Similarly, the SFR10 increases linearly ($R^2=0.998$) from 1.17 to 1.60 lp/mm.

While this improvement is consistent with the reduction in imaging skin thickness - leading to decreased light scattering and enhanced spatial frequency response - he magnitude of the change, particularly the significant increase in effective resolution, warrants further investigation.

### Flat imaging

**Fig. 9:**
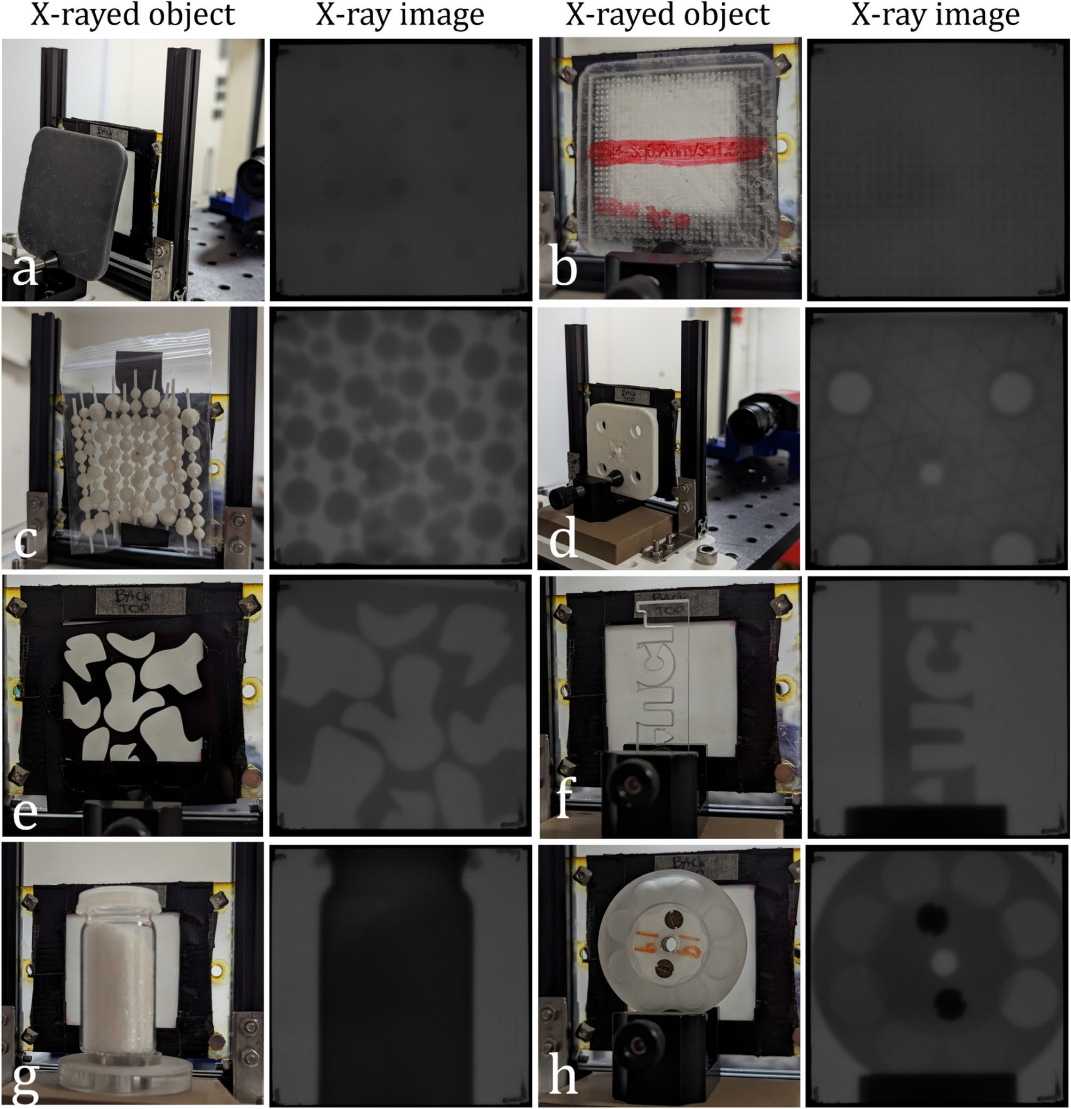
X-ray imaging. (**a**–**h**) Left image. X-rayed object (Tube voltage: 70 kVp, Exposure: 100ms). Right image. Uncorrected X-ray image. See Table 4 for more information about the material.

We experimentally measured the resolution of the sensor, but producing X-ray images was also necessary to ensure feasibility. In particular, we wanted to verify that the attenuation was sufficient for creating medical phantoms using basic prototyping materials. Therefore, Fig. 9a–h presents images obtained from imaging various objects, made of various materials (See Table 4 for details). We were pleasantly surprised by the high quality of the X-ray images, which reproduce fine details of each object. In Fig. 9d, the triangles created with Fused Deposition Modelling (FDM) at 30% infill using an Ultimaker S3 (Ultimaker B.V., Utrecht, Netherlands) 3D printer are visible. Similarly, in Fig. 9h, circles with different contrast levels, corresponding to varying filling heights, are easily discernible. By demonstrating the feasibility of capturing attenuation from prototyping materials, this confirms their suitability for constructing 3D X-ray medical phantoms and lays the foundation for the next phase of research–exploring imaging on curved surfaces.

**Table 4:** Description of materials.

Index	Material	Description
a	Tough resin (SLA^1^–Formlabs)	Round column grid ($\varnothing$3 mm, 10 mm spacing)
b	Clear resin (SLA^1^–Formlabs)	Square column grid (0.7 mm sides, 1.6 mm spacing)
c	PLA (FDM^2^–UltiMaker)	Inserts with various spheres diameter ($\varnothing$1–3.5 mm)
d	PLA (FDM^2^–UltiMaker)	3D printed square piece (30% infill)
e	Acrylic (Laser cut)	Abstract shapes
f	Acrylic (Laser cut)	UCL logo
g	Unknown	Crystals in a plastic tube (likely salt crystals)
h	Unknown	Round wheel with columns of different heights ($\varnothing$9 mm)

^1^*SLA* stereolithography, ^2^*FDM* fused deposition modelling

## Conclusion

The paper presented the design of a novel stretchable X-ray detector composed of silicone elastomer and GOS:Tb called *imaging skins*. We developed an optical stack consisting of an X-ray source, the imaging skin, and a scientific camera to collect X-ray images with this novel sensor. This research lays the foundation for the next generation of stretchable X-ray detectors, demonstrating the feasibility of an X-ray imaging detection system. By enabling direct, organ-conforming imaging, it introduces a new approach for real-time, high-resolution intraoperative imaging, where stretchable detectors could enhance tumour visualisation and improve surgical precision in minimally invasive procedures. This would reduce the reliance on pre- and post-operative imaging, potentially leading to more efficient interventions. Additionally, this specific study serves as a first step towards understanding how fabrication parameters influence sensor response in this context, guiding future developments towards clinically viable solutions.

However, the optimal design specifications for the imaging skin remain unclear. The initial hypothesis suggested that increasing thickness and phosphor concentration would enhance the final light output. However, experimentation revealed that the interplay between X-ray photon energy, phosphor concentration, and sensor thickness might affect light reabsorption by the imaging skin along with its stopping power, that would need further evaluation. Nonetheless, since increasing sensor thickness would pose challenges in a clinical setting, the 0.5 mm thickness appears to be an ideal trade-off between achieving high-quality images and ensuring ease of handling.

We also explored the impact of stretching the scintillator on imaging performance. We found that the light output of the sensor is primarily influenced by the X-ray dose received. When the sensor thickness is below the X-ray absorption limit, stretching reduces the light output by further thinning the sensor and decreasing the phosphor concentration. Conversely, if the thickness exceeds this limit, stretching enhances the light output up to the absorption threshold by reducing the self-absorption of the scintillating light. Regarding the resolution, stretching the imaging skin improved the spatial frequency resolution. Again, this improvement can be attributed to the thinning of the imaging skin caused by stretching, as it reduces light scattering, thereby enhancing the image resolution.

Finally, we imaged various objects made from different materials, focusing on those commonly used in 3D prototyping, such as acrylic, PLA and resin for 3D printing. The results are highly encouraging, and the current design and capabilities of the imaging skins suggest further research is warranted.

Although our study focused on the performance of the detector in a flat configuration, future research must explore its performance on more complex geometries. Understanding how imaging is affected when the imaging skins conform to different shapes will be crucial to obtaining accurate X-ray images with them. For example, it will be important to study how the distance between the X-ray source and the imaging skins influences the imaging quality, as conforming to a shape may cause some parts of the imaging skin to be closer to either the X-ray source or the camera. Additionally, with a fixed-focus camera lens, image distortion or blurring may occur if parts of the imaging skin lie outside the focal plane of the camera. Addressing these challenges may require programming adjustments or using a robot to position the camera around the imaging skin to capture the entire image accurately. Such understanding will be key to integrating the imaging skins effectively in surgery.

## Supplementary Information

Supplementary Information.

## Data Availability

The authors declare that the data supporting the findings of this study are available within the paper and its supplementary information files. Additional datasets, including the images generated during this study, are not publicly available due to their size and associated sharing challenges. However, these datasets, as well as the software code used in the study, can be made available by the corresponding author upon reasonable request.
