# Supplementary material for: Image quality evaluation of imaging skins, a novel stretchable X-ray detector for intraoperative tumour imaging
Source: Sci Rep. 2025 Apr 11;15:12371. doi: 10.1038/s41598-025-96768-z (PMC11985977; doi:10.1038/s41598-025-96768-z)
Supplement: Supplementary file 1 — Supplementary Information. [file 41598_2025_96768_MOESM1_ESM.pdf]

Supplementary information

Scientific Camera and phosphor specifications

X-RAY PHOSPHOR - TYPE UKL65/L-R1

GADOLINIUM OXYSULPHIDE : TERBIUM

Gd_2O_2S : Tb

PHYSICAL PROPERTIES

Material Density, g/ml : 7.5

Particle size distribution - by Coulter Counter (100 µm Aperture)

Ultrasonic Dispersion. Sizes at listed Volume %

vol %	5	25	50	75	95
µm	10.1	18.1	25.0	31.4	42.6

Quartile Deviation: 0.27

OPTICAL PROPERTIES

Emission colour : Green

Wavelength at peak, nm : 544

CIE Colour Co-ordinates : x=0.328, y=0.537

Decay Classification : Medium

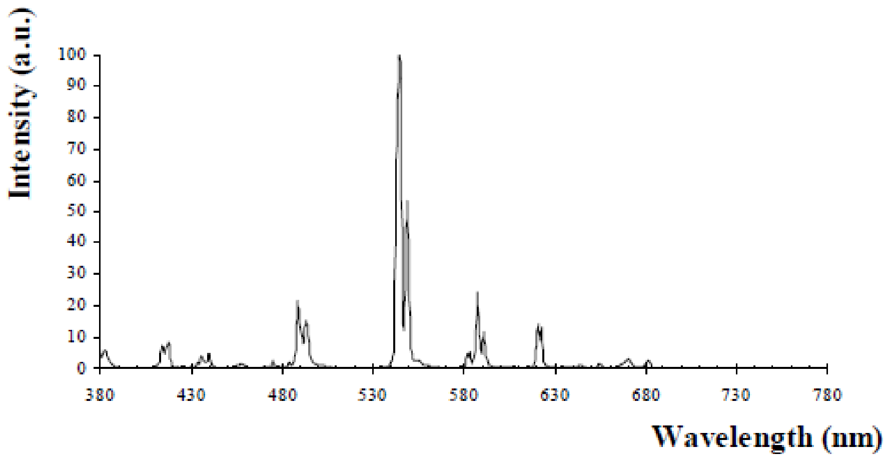

Figure S1. Supplementary material: Scintillator technical datasheet.

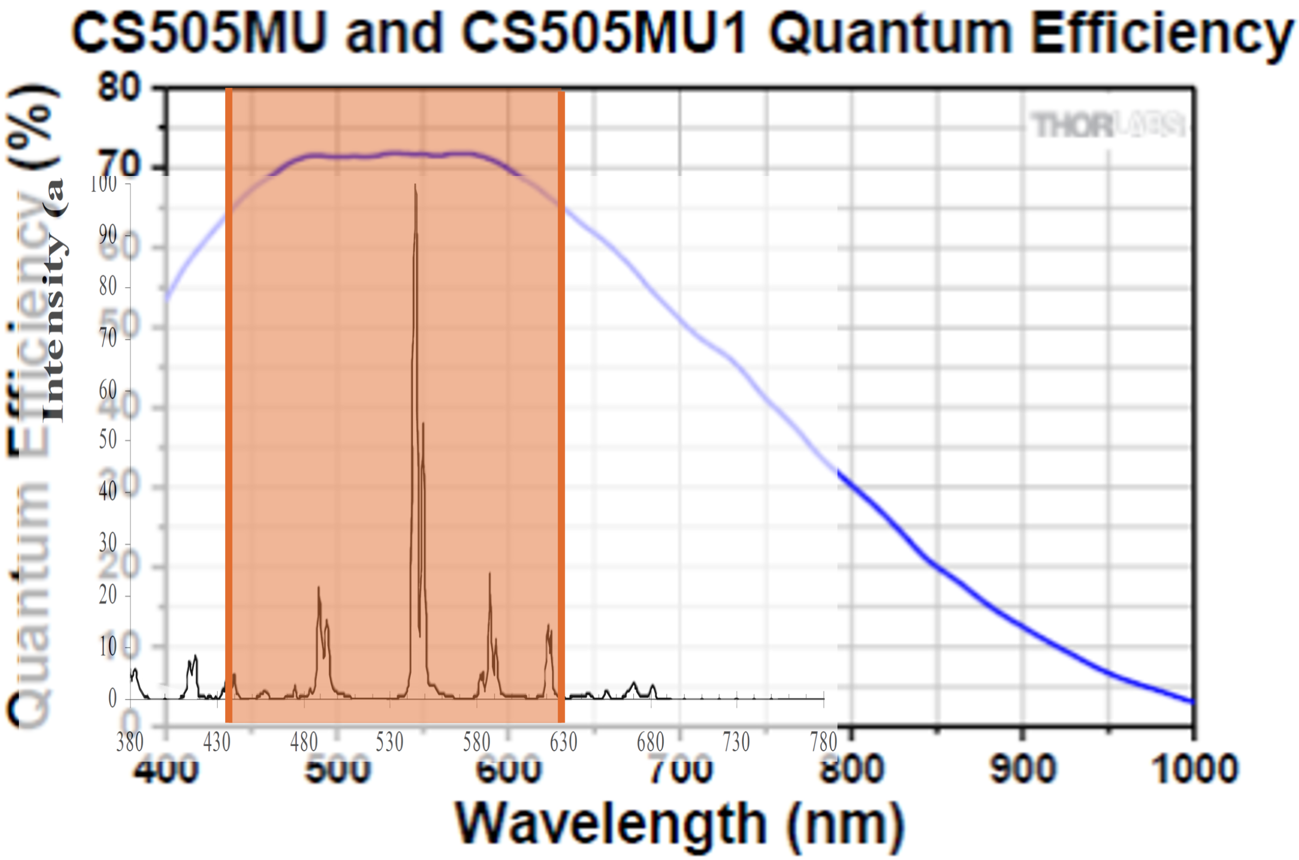

Figure S2. Supplementary material: Superposition of the CS505MU quantum efficiency and the scintillator (GOS:Tb) emission spectrum.

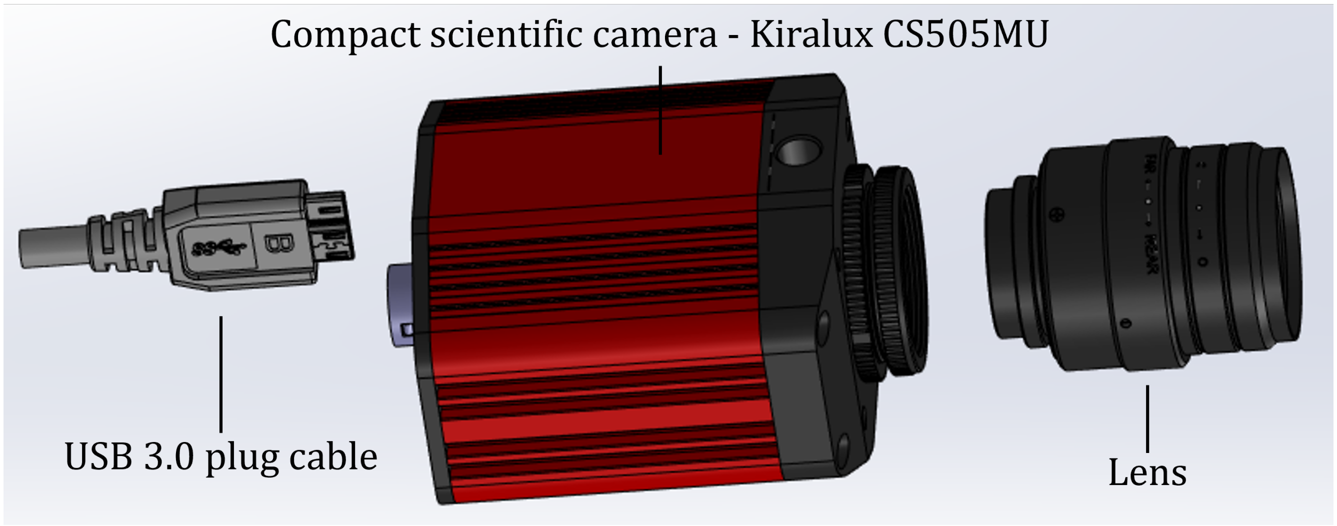

Figure S3. Supplementary material: Camera exploded view

Algorithm

Algorithm 1 Image Pre-processing

Require: Raw image I_{raw}, Flat image I_{flat}, Dark image I_{black}

$I_{\text{raw\_sub}} \leftarrow I_{\text{raw}} - I_{\text{black}}$

$I_{\text{flat\_sub}} \leftarrow I_{\text{flat}} - I_{\text{black}}$

$Mask \leftarrow \text{DetectImagingSkinContour}(I_{\text{raw\_sub}})$

$m \leftarrow \text{mean}(I_{\text{flat\_sub}}[Mask])$

$P \leftarrow I_{\text{raw\_sub}} / I_{\text{flat\_sub}} \times m$

return P

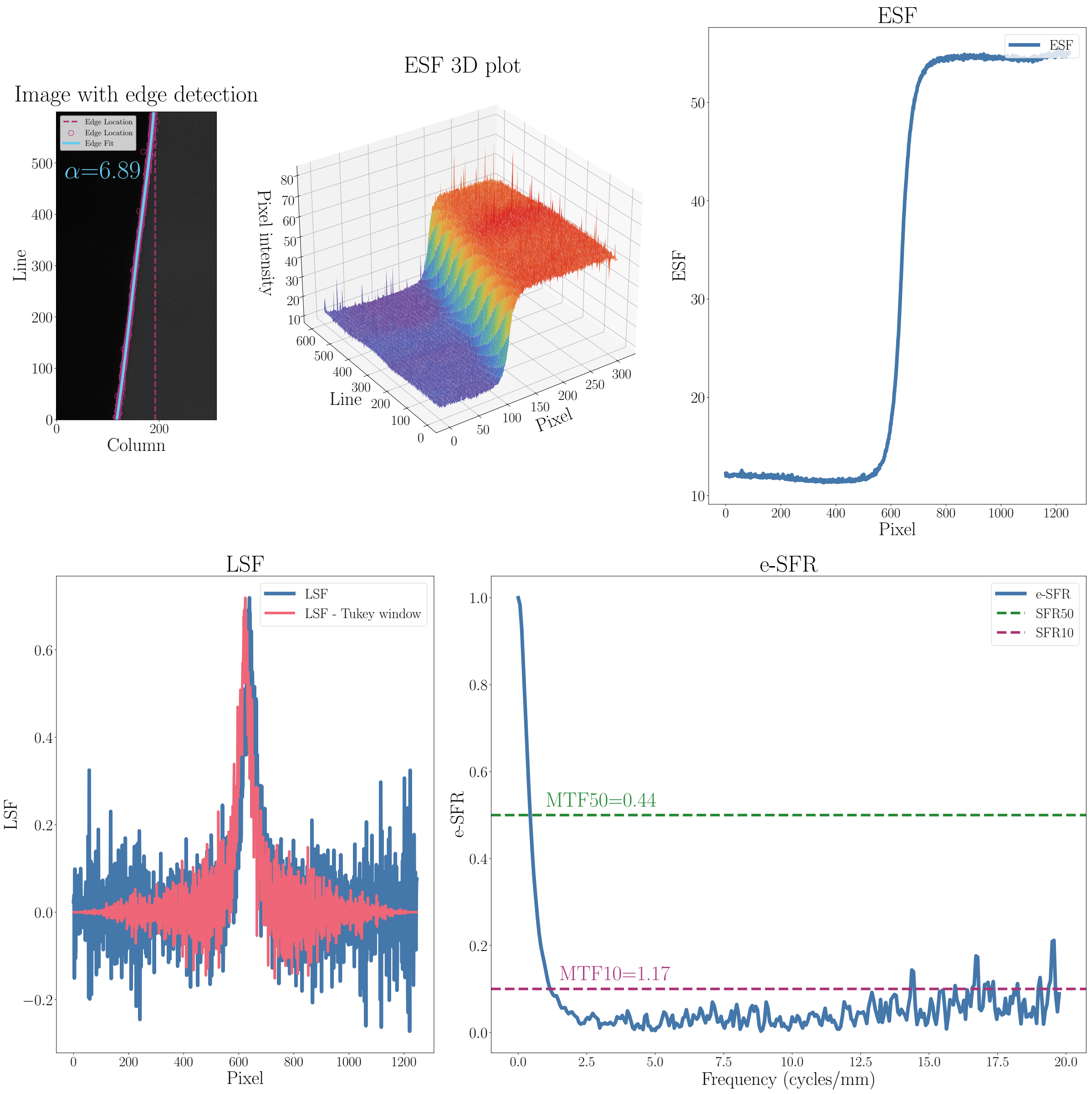

Figure S4. **SFR5MAT algorithm steps**: **(a)** X-ray image of the lead bar test pattern with the fitted edge in cyan. **(b)** 3D plot of the Edge Spread Function (ESF) for each line. **(c)** Final ESF after processing. **(d)** Line Spread Function (LSF) obtained by differentiating the ESF and applying a Tukey window. **(e)** e-SFR with SFR50 and SFR10.

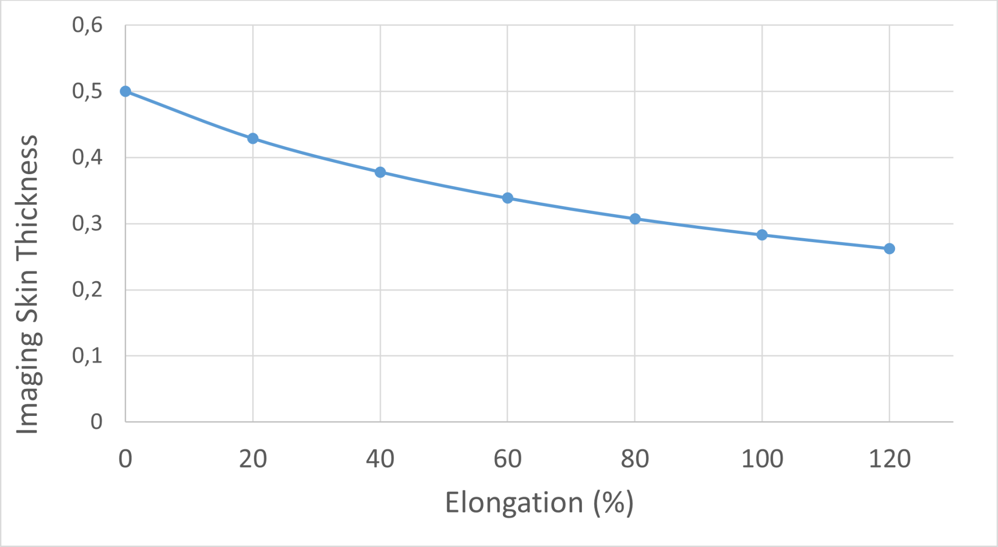

Figure S5. Estimated Imaging Skin Thickness Evolution during Stretching. The thickness was estimated by measuring the area of the imaging skin during stretching. Assuming that the volume of the imaging skin remains conserved, the thickness was inferred accordingly.

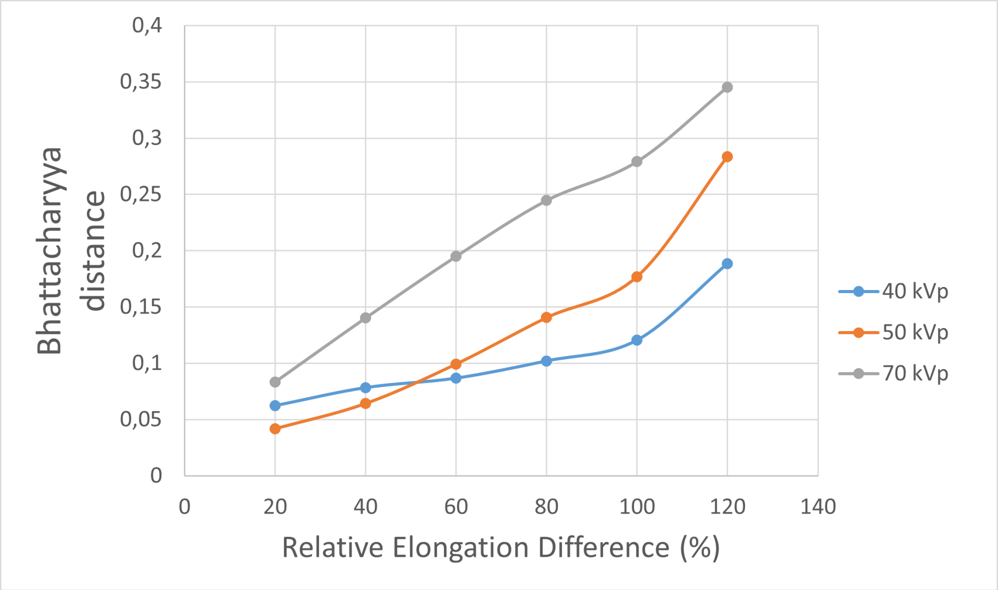

Figure S6. Evolution of the Bhattacharya distance relative to the Pixel Distribution.

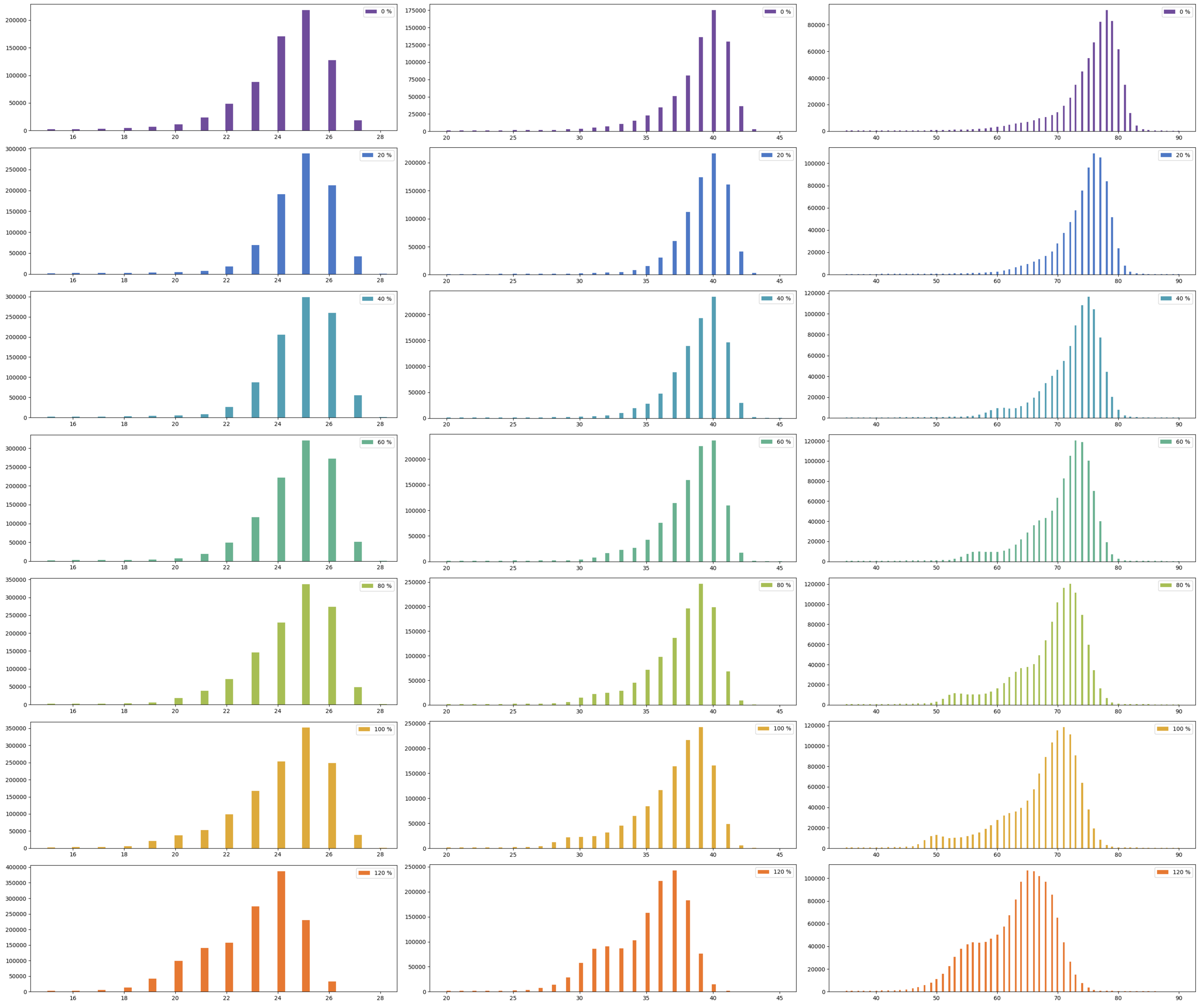

Figure S7. Pixel distribution with stretching.

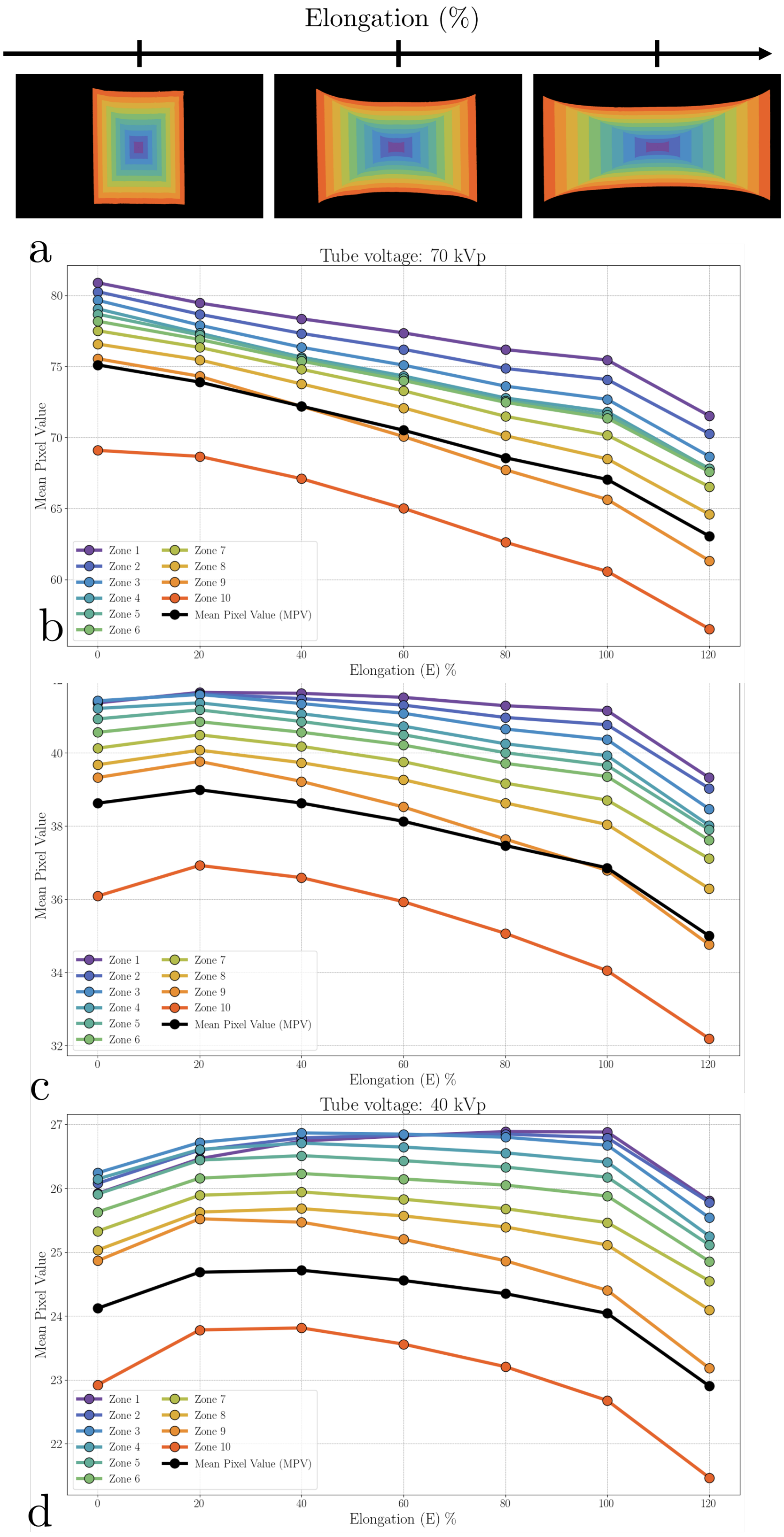

Figure S8. Local evolution during stretching.
